# Abdominal vagus nerve stimulation alleviates collagen-induced arthritis in rats

**DOI:** 10.3389/fnins.2022.1012133

**Published:** 2022-11-21

**Authors:** Sophie C. Payne, Evange Romas, Tomoko Hyakumura, Fenella Muntz, James B. Fallon

**Affiliations:** ^1^Bionics Institute, East Melbourne, VIC, Australia; ^2^Medical Bionics Department, University of Melbourne, Parkville, VIC, Australia; ^3^Department of Rheumatology, St. Vincent’s Hospital Melbourne, Fitzroy, VIC, Australia; ^4^Experimental Sciences Medical Unit, St. Vincent’s Hospital Melbourne, Fitzroy, VIC, Australia; ^5^Department of Otolaryngology, University of Melbourne, Parkville, VIC, Australia

**Keywords:** electroceutical therapy, peripheral nerve stimulation, inflammatory disease, medical devices, bioelectric medicine, vagus nerve stimulation, rheumatoid arthritis

## Abstract

Rheumatoid arthritis (RA) is a chronic, autoimmune inflammatory disease. Despite therapeutic advances, a significant proportion of RA patients are resistant to pharmacological treatment. Stimulation of the cervical vagus nerve is a promising alternative bioelectric neuromodulation therapeutic approach. However, recent clinical trials show cervical vagus nerve stimulation (VNS) was not effective in a significant proportion of drug resistant RA patients. Here we aim to assess if abdominal vagus nerve stimulation reduces disease severity in a collagen-induced arthritis (CIA) rat model. The abdominal vagus nerve of female Dark Agouti rats was implanted and CIA induced using collagen type II injection. VNS (1.6 mA, 200 μs pulse width, 50 μs interphase gap, 27 Hz frequency) was applied to awake freely moving rats for 3 h/day (days 11–17). At 17 days following the collagen injection, unstimulated CIA rats (*n* = 8) had significantly worse disease activity index, tumor necrosis factor-alpha (TNF-α) and receptor activator of NFκB ligand (RANKL) levels, synovitis and cartilage damage than normal rats (*n* = 8, Kruskal–Wallis: *P* < 0.05). However, stimulated CIA rats (*n* = 5–6) had significantly decreased inflammatory scores and ankle swelling (Kruskal–Wallis: *P* < 0.05) compared to unstimulated CIA rats (*n* = 8). Levels of tumor necrosis factor-alpha (TNF-α) remained at undetectable levels in stimulated CIA rats while levels of receptor activator of NFκB ligand (RANKL) were significantly less in stimulated CIA rats compared to unstimulated CIA rats (*P* < 0.05). Histopathological score of inflammation and cartilage loss in stimulated CIA rats were no different from that of normal (*P* > 0.05). In conclusion, abdominal VNS alleviates CIA and could be a promising therapy for patients with RA.

## Introduction

Rheumatoid arthritis (RA) is a chronic, progressive inflammatory disease that affects nearly 1.3 million people in the United States and is characterized by inflammation in the synovial joints, which leads to cartilage degradation, bone destruction, pain, and disability (McInnes and Schett, 2011; Hunter et al., 2017). Standard pharmacological therapies usually include methotrexate (first line therapy) and biological (second line) therapies such as tumor necrosis factor (TNF) inhibitors, which are expensive and cost an average of US dollars $40,000–$50,000/year (Lee et al., 2017). Despite these treatments, 20% of patients fail methotrexate intervention and 30% fail biological therapy (Bystrom et al., 2017), leading to a significant reduction in quality of life and shortened lifespan (McInnes and Schett, 2011). As such, there is a clinical unmet need for an alternative therapy for patients with RA.

The use of electricity to alter the activity of peripheral nerves, dubbed “electroceutical therapy,” has the potential to treat inflammatory diseases that are poorly controlled by pharmaceutical drugs (Waltz, 2016; Payne et al., 2019a). A growing body of evidence shows electrical stimulation of the cervical vagus nerve is a promising alternative therapy of RA (Pavlov et al., 2020). In a rat model of collagen-induced arthritis (CIA), cervical vagus nerve stimulation (VNS: delivered 60 s/day at 3 mA, 200 μs pulse width, 50 μs interphase gap, 10 Hz frequency) reduced ankle swelling and overall histological score of arthritis (inflammation, pannus formation, bone erosion, and cartilage destruction) (Levine et al., 2014). In an open label clinical trial, drug-resistant RA patients (*n* = 17; Clinical Trial ID: NCT01552941) were treated with cervical VNS (delivered 1 min once a day or 1 min four times a day at 10 Hz, 250 μs pulse width, up to 2 mA) over a 3-month treatment period. Approximately 70% of patients responded to treatment and experienced decreases in swollen joint counts, physical functional measures and key inflammatory cytokines, while withdrawal of therapy in these patients worsened the severity of the disease (Koopman et al., 2016). Resumption of VNS resulted in sustained improvements in symptoms after 2 years (Koopman et al., 2018). A recent double blinded, multi-center sham controlled study (NCT03437473) used a new generation miniaturized cervical VNS device (SetPoint Medical, Valencia, CA, United States). Only 5 out of 10 VNS (delivered 1 min once a day or 1 min four times a day at 10 Hz, 250 μs pulse width, up to 2.5 mA) treated multi-drug refractory RA patients responded, according to the European Alliance of Associations for Rheumatology (EULAR) response, and achieved reduced disease activity after 12 weeks (Genovese et al., 2020). As such, although cervical VNS is a promising treatment a significant proportion of drug-resistant patients fail to achieve low disease activity (Koopman et al., 2016; Genovese et al., 2020).

Targeting the vagus nerve at the cervical level has several limitations that could impact on the effectiveness of therapy. The cervical vagus nerve is mixed with 80% C-fibers and 20% low electrical threshold A- and B- fibers (Bonaz et al., 2013), stimulation of which often inadvertently causes activation of the larynx, heart and lungs, in addition to higher threshold C-fibers. C-fibers are at least one key neural population involved in mediating the anti-inflammatory therapeutic pathway (Martelli et al., 2014; Komegae et al., 2018; McAllen et al., 2018; Payne et al., 2018), although other neural types in the cervical vagus nerve may also have a role (Olofsson et al., 2015; Kressel et al., 2020). Previously, we have shown cervical VNS sufficient enough to evoke C-fiber neural activity in rats (10 Hz, 1.6 mA, 200 μs pulse-width) causes a severe decrease in heart and respiration rate (McAllen et al., 2018; Payne et al., 2019b). Similarly, patients can initially report voice alterations and coughing (activation of the larynx) as well as cardiac and respiratory side effects during cervical VNS (Zaaimi et al., 2005, 2007; Ben-Menachem et al., 2015). Even though stimulation-induced off-target effects can diminish over time (Ben-Menachem, 2002), it is possible they limit the amount of electrical charge that some patients can tolerate at this stimulation site. This in turn could compromise the therapeutic stimulation window that can be delivered, leading to a reduction in treatment efficacy.

Recruiting the nerve fiber population involved in activating therapeutic mechanisms is essential for an effective electroceutical treatment (Payne et al., 2019a). Applying stimulation to the abdominal vagus nerve overcomes limitations encountered during cervical stimulation. At the abdominal level, the vagus of humans consists of 99% C-fibers (Hoffman and Schnitzlein, 1961) and are distal to vagal branches that innervate the heart and lungs, which suppress respiration and heart rate during stimulation in rats (Payne et al., 2019b). As such, greater stimulation applied to the abdominal vagus nerve are well tolerated in rats (Payne et al., 2020) and could potentially permit a greater therapeutic stimulation window. We have previously demonstrated our approach of stimulating the abdominal vagus nerve is effective in relieving intestinal inflammation in a rat model of inflammatory bowel disease (Payne et al., 2019b). At present, the safety and efficacy of abdominal VNS is now being investigated in an open label interventional study for the treatment of Crohn’s Disease (ElectRx Study: NCT05469607).

Here we used the CIA model in female Dark Agouti rats (Sims et al., 2004) to assess the efficacy of abdominal VNS (1.6 mA, 200 μs pulse width, 50 μs interphase gap, 27 Hz frequency) applied for 7 days for a duration of 3 h/day. Disease activity index, including motor function, inflammation of the hind paws and swelling of ankles, was assessed and histopathology used to determine inflammation and cartilage damage.

## Materials and methods

### Animals and anesthesia

This study used a total of 24 female Dark Agouti rats (8–9 weeks old, Animal Resource Centre, Murdoch, WA, Australia). All procedures were approved by the Animal Ethics Committee of St. Vincent’s Hospital (Melbourne) and complied with the Australian Code for the Care and Use of Animals for Scientific Purposes (National Health and Medical Research Council of Australia) and the Prevention of Cruelty to Animals (1986) Act. Implanted rats were housed individually with environmental enrichment under a 12 h light/dark cycle and allowed *ad libitum* access to standard chow and water. For surgical interventions, rats were anesthetized (2–3% isoflurane, 1–1.5 L/min oxygen) and given an analgesic (Carprofen 5 mg/kg sub-cutaneous). At the end of the experiment, rats were anesthetized (2% isoflurane using an oxygen flow rate of 1–1.5 L/min), final electrophysiology tests performed and then euthanized (300 mg/kg Lethabarb, intraperitoneal injection).

### Abdominal vagus nerve array and implantation surgery

**FIGURE 1:**
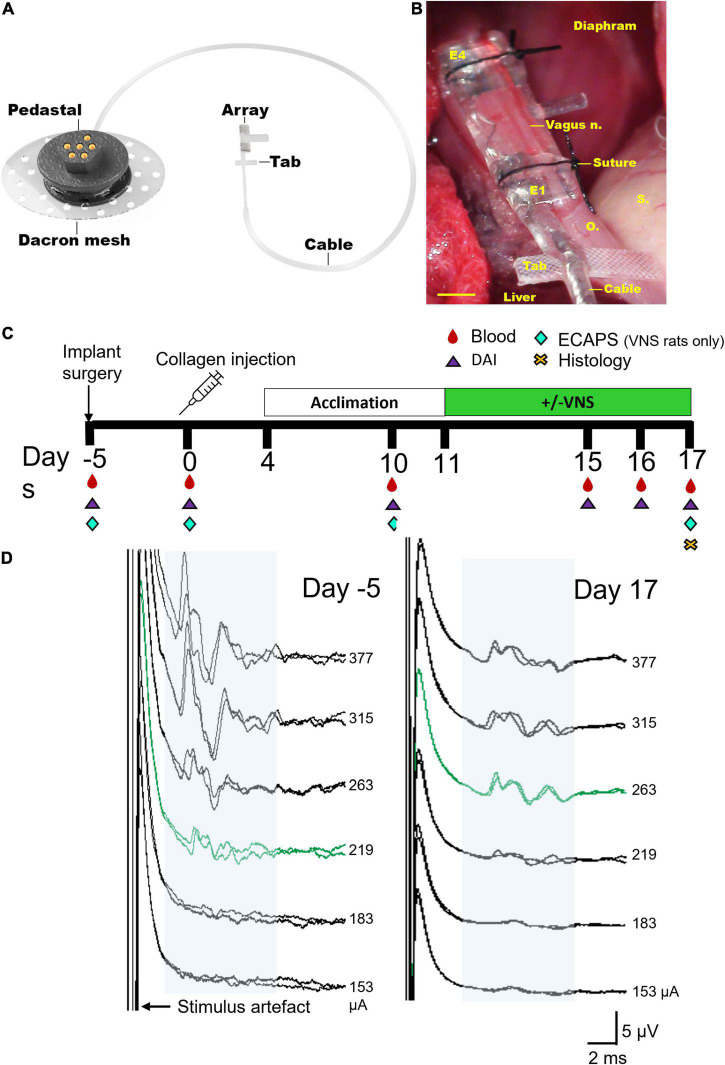
Electrode array and experimental schedule. **(A)** Rendered drawing of the cuff electrode array and percutaneous pedestal. **(B)** An *in vivo* image shows the placement of the array below the diaphragm on the ventral esophagus (“o”) adjacent to the stomach (“s”) and liver. The sub-diaphragmatic anterior abdominal vagus nerve (vagus n.) was implanted and sutures used to hold the lid of the device closed. The tab was sutured to the underlying esophagus (“o”) for stability. Electrodes 1 and 2 (E1–E2) were used to deliver bipolar stimulation while electrodes 3 and 4 (E3–E4) recorded electrically evoked compound action potentials. **(C)** Experimental schedule for the chronic stimulation experiment (DAI: disease activity index, includes assessments on locomotor function, macroscopic digit inflammation and ankle swelling; ECAPs: electrically evoked compound action potentials). Collagen-induced arthritis was observed in rats on days 15–17. **(D)** Representative electrically evoked compound action potentials (ECAPs) after surgery (day -5) and before termination (day 17). Neural thresholds are indicated in green (day -5: 219 μA; day 17: 263 μA) and the neural response window in light blue shading. Current amplitude (in μA) is indicated to the right of each set of ECAPs. Scale bar in panel **(B)** represents 1 mm.

*Vagus nerve array*: A custom-designed 4-platinum electrode cuff array specifically designed for implantation onto the abdominal vagus nerve of rats was used, as previously described (Payne et al., 2019b). The electrode surface area was 0.3 mm^2^, the distance between adjacent electrodes (E1–E2, or E3–E4, center to center) was 1.2 mm and the distance between adjacent pairs of electrodes (center to center) was 4.7 mm (Figure 1A). A cable ran subcutaneously to a percutaneous pedestal that was sutured to the dorsal-lumbar aspect of the back. *Implantation surgery*: As described previously (Payne et al., 2019b), animals were anesthetised, and under aseptic conditions the abdominal cavity exposed and the sub-diaphragmatic anterior abdominal vagus nerve, rostral to the hepatic and celiac vagal branches, was identified and dissected from the esophagus. The cuff electrode was placed around the nerve and sutures tied to hold the cuff closed (Figure 1B), and the cable sutured (7-0 silk, Ethicon, WestSomerville, NJ, United States) to the underlying esophagus. The rat was rotated, and the percutaneous connector was anchored to the lumbar region of the spine and the skin closed.

### Experimental cohorts and schedule

The experimental groups consisted of a normal group (*n* = 8) that had no collagen injections or vagal implant but were handled in the same way as the implanted collagen induced arthritis (CIA) rats. Unstimulated CIA rats (*n* = 8) received an implant and a collagen injection, however, these rats were not tested for impedances or electrically evoked compound action potentials (ECAPs) to avoid potential delivery of small doses of VNS therapy. Stimulated CIA rats (*n* = 8 initially, *n* = 6 included in study) received an implant, collagen injection and were tested for impedances and ECAPs (Figure 1C). ECAPs could not be recorded in *n* = 2 of 8 rats on day 17 and so these animals were excluded from the analyzable population because the delivery of therapeutic stimulation could not be confirmed. All groups underwent acclimation handling from days 4–11 and either sham or VNS stimulation from days 11–17. Peripheral blood (300 μL) and disease activity index (DAI) was assessed on the day of surgery and on days 0, 10, 15–17 following collagen injection, and histology was taken on day 17 (Figure 1C).

### Collagen-induced arthritis model

Collagen-induced arthritis is an established model of rheumatoid arthritis (Romas et al., 2000, 2002; Corti and Ghezzi, 2004; Sims et al., 2004). Anaesthetized rats were immunized with 300 μL (6 × 50 μL) intradermal injections of bovine collagen type II (2 mg/kg, Sigma) and heat-killed Mycobacterium tuberculosis (Freund’s Solution, Sigma) into the base of the tail. After 2 weeks, evidence from previous studies show over 95% of rats reliably developed arthritis (Romas et al., 2000; Corti and Ghezzi, 2004).

### Disease activity index

The disease activity index is comprised of measurements that assess locomotor function, macroscopic inflammation of hind digits and ankle and foot swelling. Observers (SP, TH) were blinded to experimental conditions. *Functional score*: The animal’s mobility was scored (0–3), with the scoring system being: 0 = no limping, 1 = difficulties bearing weight i.e., limping on one leg, 2 = limping on 2 paws, 3 = animal experienced difficulty in moving, and favored being inert. Animals were briefly anaesthetized to ensure accurate measurements of inflammatory score and dimensional measurements. *Inflammatory score*: Hind paws were macroscopically scored (range 0–5 for each hind paw), as described previously (Romas et al., 2000). The scoring system is as follows: 0 = no arthritis, 1 = swelling and/or redness of 1–2 interphalangeal (IP) joints, 2 = involvement of 3–4 IP joints, 3 = more than 4 joints red/swollen, 4 = swelling/redness of the entire paw, and 5 = entire paw and ankle. A total score of 0 to 10 of both hind paws was possible. *Ankle and foot width measurements*: The ankle widths were measured using digital calipers. The thickness of the mid-plantar hind paw was also measured.

### Impedance testing and electrophysiological recordings

Functionality of electrodes was assessed in stimulated rats prior to delivering stimulation or electrically evoked compound action potentials (ECAP) testing by measuring common ground impedance by passing biphasic current (931 μA, 25 μs) pulses between the electrode of interest and all other electrodes. The peak voltage at the end of the first phase (Vtotal) of the current pulse was measured, and the Vtotal value was used to calculate total impedance (Ztotal) using Ohm’s law (*Z* = voltage/current) (Payne et al., 2018). ECAPs were used to determine threshold of neural activation and recorded in stimulated rats after implantation surgery and on day 17 following collagen injection (Figure 1D). ECAPs were recorded between bipolar pairs of electrodes, with electrodes 1–2 delivering stimulation and electrodes 3–4 recording the evoked response. Two sets of evoked bipolar recordings (averaged from a total of 50 responses) were made at currents from 0 to 1.6 mA in 0.1 mA steps using 10 Hz delivered at 200 μs pulse width and 50 μs interphase gap. Recordings were sampled at a rate of 100 kHz and filtered (500 Hz–3000 Hz; voltage gain 10^2^) (Payne et al., 2019b; Fallon and Payne, 2020). The ECAP threshold was defined as the minimum stimulus intensity producing a response amplitude of at least 0.1 μV within a post-stimulus latency window of 4–10 ms. Electrodes 1–2 were used to deliver VNS treatment to CIA rats.

### Habituation and vagus nerve stimulation

Rats from all groups were acclimatized in a testing cage for 7 days (days 4–11) to ensure no additional stress during the testing period. From days 11 to 17, implanted rats had 7 testing sessions in total in which stimulated cohorts received 3 h of stimulation a day (27 Hz, 1.6 mA, 200 μs pulse-width with 50 μs interphase gap, 30 s ON, 2.5 min OFF) while awake using an in-house stimulator (Figure 1C; Fallon et al., 2018). ECAPs were generated on days 10 and 17, only in the stimulation cohort, to ensure stimulation was suprathreshold (Figure 1D). Recording of ECAPs was an inclusion criterion for the stimulation cohort, and 2 out of 8 rats were excluded from the study as no ECAPs were recorded on day 17. The unstimulated cohort was plugged in, but stimulators were not switched on. Normal (i.e., non-implanted) rats were placed in testing boxes and handled in the same way as implanted cohorts.

### Molecular analysis

Whole blood was collected by cardiac puncture on day 17, collected in K2- ethylenediaminetetraacetic acid (EDTA) tubes (Starstedt, SA, Australia), centrifuged (2000 *g* for 10 min) and plasma aliquoted and stored at −80°C. On the day of the assay, aliquots were thawed on ice and the tumor necrosis factor alpha (TNF-α, Sigma, VIC, Australia) and receptor activator of nuclear factor kappa-B ligand (sRANKL, Jomar Life Research, Melbourne, VIC, Australia). Enzyme-linked immunosorbent assays (ELISAs) were conducted according to manufacturer’s instructions. Levels in plasma were determined *via* absorbance measurements using a Biorad BenchMark Plus microplate spectrophotometer (PerkinElmer, Inc., Waltham, MA, United States). The lowest level of detection (LLOD) for the TNF-α assay is 83 pg/ml, and sRANKL is: 156 pg/ml, and <LLOD indicates below this level.

### Histological processing

At the end of the experiment, animals were euthanized and perfused intracardially with 0.9% saline and fixative (10% neutral buffered formalin in 0.1 M phosphate buffer, pH 7.4, room temperature) (Keast and Osborne, 2019). The digits of left and right hind paws were dissected, post-fixed at 4°C overnight and decalcified in 10% EDTA in phosphate buffered saline (PBS) for 3 weeks until soft enough to cut with a scalpel. Digits underwent a standard 12 h overnight processing for paraffin embedding. Serial 5 μm sagittal sections of digits were taken and stained with hematoxylin and eosin (H&E) for evaluation of inflammation or 1% toluidine blue (pH 4) (Bergholt et al., 2019) for assessment of cartilage damage, and mounted with dibutylphthalate polystyrene xylene.

### Histopathological scoring and imaging

Observers (FM: veterinary pathologist; SCP; ER: rheumatologist) were blinded to experimental conditions and used H&E stained sections to evaluate the degree of inflammation within the distal-middle phalangeal joint space. Scoring of inflammation was compositive of synovitis, periarticular infiltration and osteitis. H&E and toluidine blue stained sections were used to evaluate the degree of pannus formation and cartilage damage within the distal-middle phalangeal joint space. Tissue was scored according to parameters outlined in Table 1, which were adapted from previous studies (Sims et al., 2004; Levine et al., 2014). Representative light microscope images of H&E sections were taken using a Zeiss Axioplan II microscope (Carl Zeiss Microscopy, Jena, Germany) and AxioVision Software (Zeiss, Germany, New York, Unites States).

**TABLE 1:** Histological scoring of inflammation, pannus formation, and cartilage damage.

	Score	Scoring criteria
Inflammation	0 1 2 3 4	None present Mild infiltrate of neutrophils into distal to middle phalangeal joint space Moderate infiltrate of neutrophils into phalangeal joint space Moderate infiltrate of acute inflammatory cells (neutrophils/eosinophils), and mild appearance of chronic inflammatory cells (lymphocytes, macrophages/synoviocytes) in joints and adjacent soft tissues (ligament, tendon, digital pad) and edema Severe infiltrate of acute inflammatory cells and mild appearance of chronic inflammatory cells in joints and adjacent soft tissues and edema
Pannus formation	0 1 2 3	None present Minimal infiltration (<1/4 of tibia edges) of pannus in cartilage and subchondral bone Moderate infiltration (1/4< >1/2 tibia edges) Severe infiltration (1/2<tibia edges)
Cartilage damage	0 1 2 3 4 5	None present Minimal loss of toluidine blue staining Mild loss of toluidine blue staining and mild thinning Moderate loss of toluidine blue staining to mid zone Marked loss of toluidine blue staining to deep zone Severe loss of toluidine blue staining to tide mark

### Statistics and figure production

Differences between normally distributed data was analyzed using parametric one-way analysis of variance (ANOVA) or *t*-tests. Non-normally distributed data was analyzed using a non-parametric Kruskal–Wallis one-way ANOVA and Dunn’s *post-hoc* test. Statistically significant differences were accepted as *P*-values of < 0.05 and GraphPad Prism 4 (GraphPad Software, United States) was used for all analysis. For figure production, light microscope images were white color balanced and adjustments made where necessary in contrast and brightness to best represent that seen under the microscope (Adobe InDesign and Photoshop CS6; Adobe Systems, San Jose, CA, USA).

## Results

### Electrically evoked compound action potential thresholds

Electrically evoked compound action potentials (ECAPs) were recorded in collagen induced arthritis (CIA) rats randomly selected to receive VNS treatment (representative traces in Figure 1D). Immediately after surgery the neural threshold of rats in the stimulated CIA group (*n* = 8) was 252 ± 34 μA (range: 128–377 μA) and was recorded at an average latency of 6.5 ± 0.9 ms, which generated an approximate average conduction velocity of 0.80 ± 0.08 m/s (range: 0.39–1.12 m/s). At day 17 there were no significant changes in in threshold (667 ± 144 μA, range: 153–1114 μA, *P* = 0.051, *n* = 6, student paired *t*-test between responding rats). The average latency of recorded responses was 5.9 ± 0.4 ms, which generated an approximate average conduction velocity of 0.81 ± 0.05 m/s (range: 0.61–0.99 m/s). ECAPs could not be recorded in *n* = 2 rats on day 17 and so these CIA rats were excluded from the analyzable population because the delivery of therapeutic stimulation could not be confirmed.

Common ground impedance of the stimulation CIA group immediately after surgery was 8.0 ± 0.6 kOhms and significantly increased to 15.5 ± 1.3 kOhms at day 17 (student paired *t*-test between responding rats, *n* = 6, *n* = 24 electrodes, *P* = 0.005). All electrodes in the stimulated cohort (*n* = 6) remained functional and there were no open or short circuits.

Rats in the stimulation CIA (VNS, *n* = 6) group were stimulated while awake and freely moving for an average total of 19.8 ± 0.8 h or 2.8 h/day over 7 continuous days. Unstimulated CIA rats (*n* = 8) were tethered to stimulators (switched off) for an average total of 20.9 ± 1.3 h or 3.0 h/day over 7 continuous days. There were no significant differences between tethering time of stimulated and unstimulated CIA rats (unpaired student *t*-test, *P* = 0.60).

### Effects of vagus nerve stimulation on measurements of disease activity index

**FIGURE 2:**
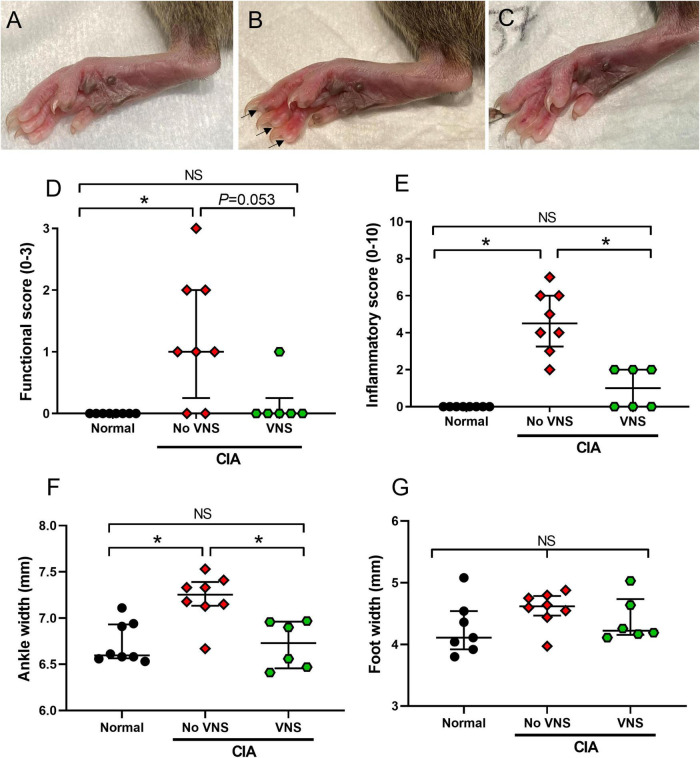
Effects of vagus nerve stimulation improves on measures of disease activity index at 17 days following collagen injection. **(A–C)** Representative images of hind paws from normal **(A)**, unstimulated collagen-induced arthritis (CIA) [**(B)**, no vagus nerve stimulation (VNS), inflamed digits indicated by arrows], and stimulated CIA [**(C)**, VNS] rats at 17 days following a collagen injection. **(D)** Limping was assessed by measuring functional score. **(E)** Inflammation of the paws was assessed by measuring inflammatory score. **(F,G)** Swelling of hind paws was assessed by measuring ankle width **(F)** and foot width **(G)**. Data show median ± interquartile range and symbols are values from individual rats. Differences between normal (*n* = 7–8), no VNS (*n* = 8), and VNS (*n* = 6) groups was analyzed using Kruskal–Wallis one-way ANOVA and Dunn’s *post-hoc* test. Significant differences of *P* < 0.05 are indicated by “*” and non-significant differences as “NS”.

The final disease activity index (functional score, inflammatory score, ankle and foot width measurements) of rats was analyzed at 17 days following the collagen injection. Disease activity index data was not normally distributed, therefore significant differences were assessed using a non-parametric Kruskal–Wallis one-way ANOVA and a Dunn’s *post-hoc* test. Representative images show the hind paw of a normal (Figure 2A) rat, as well as an unstimulated (no VNS, Figure 2B) and stimulated (VNS, Figure 2C) rat.

*Functional score*: Following the induction of CIA, functional score significantly increased in unstimulated CIA rats (No VNS) compared to normal rats (ANOVA: *P* = 0.004, Dunn’s *post-hoc* test: *P* = 0.005, *n* = 8, Figure 2D). In stimulated CIA rats (VNS) there was a decrease in functional score compared to unstimulated CIA rats. Although this decrease failed to reach significance (Dunn’s *post-hoc* test: *P* = 0.053, *n* = 6), functional score in stimulated CIA rats was no different to normal (Dunn’s *post-hoc* test, *P* > 0.999, *n* = 8, Figure 2D). A chi-square test of independence was performed to examine the relation between treatment and limping behavior following a collagen injection. The relation between these variables was significant, *X*^2^ = 4.67, *p* = 0.0154, suggesting unstimulated CIA rats were more likely to limp than stimulated CIA rats.

*Inflammatory score*: Unstimulated CIA rats had a significantly higher inflammatory score in hind paws compared to normal rats (ANOVA: *P* < 0.0001; Dunn’s *post-hoc* test: *P* = 0.0001, *n* = 8, Figure 2E). However, in stimulated CIA rats inflammatory score significantly decreased compared to unstimulated CIA rats (*P* = 0.026, *n* = 6, Figure 2E).

*Ankle and foot width measurements*: The ankle diameter of unstimulated CIA rats significantly increased compared to normal rats (ANOVA: *P* = 0.002, Dunn’s *post-hoc* test: *P* = 0.006, *n* = 8). However, the ankle diameter of stimulated CIA rats significantly decreased compared to unstimulated CIA rats (Dunn’s *post-hoc* test: *P* = 0.011, *n* = 6, Figure 2F). There were no changes to the width of the hind paw following the CIA injection (ANOVA: *P* = 0.19; Figure 2G) compared to normal (*n* = 7).

### Effects of vagus nerve stimulation reduces on plasma levels of tumor necrosis factor-alpha and soluble receptor activator of NFκB ligand

Levels of TNF-α in plasma remained below the lowest level of detection (LLOD) for this assay (83 pg/ml) in normal rats (*n* = 8). At 17 days following collagen injection, plasma TNF-α in unstimulated CIA rats (*n* = 8) was elevated at 511 ± 231.3 pg/ml. However, levels of plasma TNF-α were below the lowest level of detection (83 pg/ml) in stimulated CIA rats (*n* = 6, Table 2).

Levels of soluble receptor activator of NFκB (sRANKL) in plasma of normal rats (*n* = 8) were below the lowest level of detection (156 pg/ml). At 17 days following collagen injection, levels of sRANKL in plasma increased to 357 ± 31.8 pg/ml in unstimulated CIA rats, but were significantly decreased (237 ± 9.7 pg/ml) in stimulated CIA rats (unpaired student *t*-test, *P* = 0.045).

### Effects of vagus nerve stimulation on histological measures of inflammation and joint damage

**FIGURE 3:**
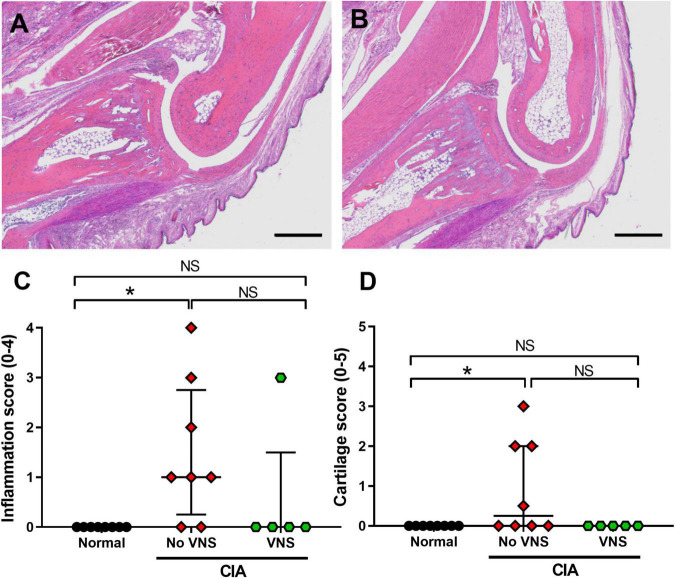
Effects of vagus nerve stimulation improves histopathology of interphalangeal joints at 17 days following collagen injection. **(A)** Representative image of inflammation in and around the distal interphalangeal (IP) joint from an unstimulated collagen-induced arthritis (CIA) rat shows moderate neutrophil infiltration. **(B)** The IP joint of stimulated CIA rats had little to no inflammation within the joint space. **(C)** Semi-quantification of inflammation histological score (0–4) using H&E staining. **(D)** Semi-quantification of cartilage damage (0–5). Data show median ± interquartile range and symbols are from individual rats. Differences between normal (*n* = 8), no vagus nerve stimulation (VNS) (*n* = 8), and VNS (*n* = 5) groups was analyzed using Kruskal–Wallis one-way ANOVA and Dunn’s *post-hoc* test. Significant differences of *P* < 0.05 are indicated by “*” and non-significant differences as “NS.” Scale bars in panel **(A)** and **(B)** are: 500 μm.

*Inflammation*: Qualitative observations made by three observers blinded to treatment (FM: veterinary pathologist; SCP; ER: rheumatologist) show normal animals had no infiltration of neutrophils within the distal interphalangeal joint space or surrounding tissues. At 17 days following collagen injection, unstimulated CIA rats were observed to have mild infiltration of neutrophils into the distal interphalangeal joint space (Figure 3A) and low levels of synovitis and periosteal inflammation. However, in the majority of distal interphalangeal joints (4 of 5 samples) taken from stimulated CIA rats there were no signs of acute or chronic inflammatory cell infiltration (Figure 3B). Semi-quantification of histological inflammation shows scores were significantly higher in unstimulated CIA rats (No VNS, Kruskal–Wallis ANOVA: *P* = 0.011; Dunn’s *post-hoc*: *P* = 0.009, *n* = 8) compared to normal rats (Normal, *n* = 8; Figure 3C). Although there was no difference in inflammation score between unstimulated and stimulated CIA rats (*P* = 0.223), score in stimulated CIA rats (VNS, *n* = 5) was no different to that seen in joints of normal rats (Dunn’s *post-hoc*: *P* > 0.999, Figure 3C).

*Pannus formation*: Histopathological assessment showed pannus formation was only observed in 1 of 8 unstimulated CIA rats (Score: 1), which had the most severe arthritis. No pannus formation was seen in normal or stimulated CIA cohorts.

*Cartilage damage*: There was a minimal but significant increase in the cartilage score from unstimulated CIA rats (Kruskal-Wallis ANOVA: *P* = 0.031), compared to normal tissue (Dunn’s *post-hoc* test: *P* = 0.041, *n* = 8, Figure 3D). Although there was no difference in cartilage score between unstimulated and stimulated CIA joints (*P* = 0.091), scores from stimulated CIA rats were not significantly different from that of normal rats (*P* > 0.9999; Figure 3D).

**TABLE 2:** Effect of vagus nerve stimulation on tumor necrosis factor-alpha (TNF-α) and soluble receptor activator of NFκB ligand (sRANKL) levels at 17 days following collagen injection.

Cohort	TNF-α (pg/ml)	sRANKL (pg/ml)
Normal	<LLOD	<LLOD
Unstimulated CIA (no VNS)	511 ± 231.3	357 ± 31.8*
Stimulated CIA (VNS)	<LLOD	237 ± 9.7

Data are means ± SEM of *n* = 8 normal, *n* = 8 unstimulated collagen-induced arthritis (CIA), n = 6 CIA stimulated rats. An unpaired student *t*-test was used to compare between TNF-α or sRANKL levels in no vagus nerve stimulation (VNS) vs. VNS groups and ‘*’ indicated *P* < 0.05. Lowest level of detection (LLOD) for the TNF-α assay is 83 pg/ml, and sRANKL is: 156 pg/ml, and <LLOD indicates below this level.

## Discussion

Cervical vagus nerve stimulation is a promising therapy for RA, however, recent clinical trials show cervical vagus nerve stimulation (VNS) was not effective in a significant proportion of drug resistant RA patients (Koopman et al., 2016; Genovese et al., 2020). Here, we show that abdominal VNS, which is an alternative stimulation site that avoids side effects associated with cardiac and pulmonary vagus nerve projections, improves measures of collagen induced arthritis (CIA). Specifically, these include improvements in disease activity index, TNF-α and sRANKL levels, and a reduction in synovitis and cartilage damage. This study demonstrates in principle that abdominal VNS is effective and could translate into an alternative therapy to cervical VNS for patients with RA.

Absence of treatment effect in 30–50% of patients and large inter-patient variability following cervical VNS may be due to failure to activate therapeutic fibers (Waltz, 2016; Genovese et al., 2020). Abdominal VNS allows for the recording of electrically evoked neural responses (Payne et al., 2018, 2019b), which permits confirmation that stimulation is “suprathreshold” or sufficient to activate anti-inflammatory neural pathways. A major strength of our study is that all CIA rats that received abdominal VNS had confirmed neural thresholds below 1.6 mA i.e., the current level of therapeutic stimulation, and all recorded neural responses, had conduction velocities consistent with vagal mammalian C-fibers of 0.3–2 m/s (Castoro et al., 2011). We expect confirmation that stimulation is suprathreshold and will help to reduce some inter-patient variability observed during early bioelectric clinical trials (Payne et al., 2019a). Furthermore, recording evoked neural responses also permits titration of an optimal stimulation “dosage” according to the clinical response. Stimulating at levels far higher than necessary to adequately activate therapeutic fibers can produce desensitization from overstimulation, and refractoriness to VNS (Waltz, 2016). Without a measure of activity of the target fibers, there is a risk of stimulation below a threshold that generates a therapeutic effect. As such, recording the evoked neural responses is a desirable and important feature that should be included in future clinical trials.

At 17 days following the collagen injection, we report statistically significant worsening in several markers of CIA, compared to normal rats, which include measurements of the disease activity index (functional score, inflammatory score, and ankle width), systemic molecular markers and histopathology. A similar CIA model was successfully used to assess the efficacy of cervical VNS (Levine et al., 2014). Both studies resulted in moderate to marked levels of inflammation that caused ankle swelling, but minimal to mild pannus formation and cartilage damage. The rat model of CIA is a well-established model of inflammatory arthritis that was first described in the 1970s, and rapidly develops 2–3 weeks after collagen II injection to primarily cause arthritis in the interphalangeal and ankle joints of the hind paws (Trentham et al., 1977). Due to its reproducibility and similarities to human RA, CIA is commonly used to assess the efficacy of pharmacological therapies, such as methotrexate, ibuprofen, and dexamethasone (Brahn et al., 1991; Du et al., 2008; Le Goff et al., 2009; De et al., 2017; Wang et al., 2019). While CIA progresses more rapidly than human RA, it shares similar clinical and molecular mechanisms including the activation of osteoclasts by TNF-α and RANKL (Schopf et al., 2006). The pathogenesis of RA involves activation of adaptive immunity (CD4 + T cells and autoantibody producing B cells), synovial inflammation with infiltration of immune cells and release of pro-inflammatory cytokines (e.g., TNF-α) culminating in the destruction of cartilage and bone (Schopf et al., 2006; McInnes and Schett, 2011).

Abdominal VNS was effective in reducing collagen-induced-arthritis, primarily indicated by decreases in limping, redness of digits, ankle swelling. Abdominal VNS was also effective in reducing systemic TNF-α, sRANKL, and histological scores of inflammation and cartilage damage to levels no different to normal. Most notably, our results are comparable to the improvements in CIA reported following cervical VNS (Levine et al., 2014). Both the current study and Levine et al. (2014) applied VNS before the clinically apparent disease onset (i.e., on days 9 and 11 following collagen injection). Although synovitis is not macroscopically visible at this stage, activation of adaptive immunity and inflammatory process has entered the pre-effector “immune” phase of the model’s disease progression (Schopf et al., 2006). It is well established that vagus nerve stimulation activates the cholinergic anti-inflammatory pathway, mediated by efferent vagal fibers (Pavlov et al., 2003; Rosas-Ballina et al., 2011; Pavlov and Tracey, 2017). In support of this, we found elevated levels of TNF-α in plasma in unstimulated CIA animals, while levels of TNF-α in stimulated CIA animals remained below detectable levels for the assay (83 pg/ml). TNF-α is a key pro-inflammatory cytokine upregulated in RA, and pharmacological agents that block TNF-α are effective therapies for RA (Bongartz et al., 2006). We also observed less infiltration of acute inflammatory cells such as neutrophils in stimulated CIA, compared to unstimulated CIA, an observation supported by previous VNS studies (de Jonge et al., 2005; Huston et al., 2009).

In this study, the suprathreshold stimulation parameters of 1.6 mA and 200 μs pulse-width (320 NC charge) delivered using a duty cycle of 30 s ON, 2.5 min OFF was similar to that effectively used for the treatment of experimental intestinal inflammation in rats (Payne et al., 2019b). This duty cycle was historically adapted from cervical VNS clinical trials of inflammatory bowel disease, which used 30 s ON, 5 min OFF, (Bonaz et al., 2016; Sinniger et al., 2020) which in turn were based on those used to treat patients with drug-resistant epilepsy and depression (Cukiert et al., 2013). However, previous preclinical studies using cervical VNS as a treatment of CIA used 3 mA and 200 μA pulse width (600 nC charge) that was delivered for only 60 s per day. Similarly, 50–70% of patients were classified as “responders,” according to the EULAR response, and had reduced disease activity following short stimulation doses (60 s, from one to four times per day) after 12 weeks (Koopman et al., 2016; Genovese et al., 2020). The shift to use ultralow duty cycles was supported by the finding that a single 60 s pulse applied to the cervical vagus nerve was sufficient reduced systemic serum TNF levels in a rat model of sepsis (Olofsson et al., 2015). However, to date there have been no comprehensive studies investigating the effects of other stimulation regimes on either arthritis in rats or humans. Assessing stimulation dose is a vexing problem due to the high number of stimulation parameters (electrical pulse, duty cycle, frequency train, or charge) that can be changed to impact on how efficiently fiber populations are activated. Lack of investigation into bioelectric therapy “dosing” is thought to be a key factor in inter-patient variability in response to treatment and failure of clinical trials in reaching significant outcomes (Waltz, 2016; Payne et al., 2019a). Future pre-clinical studies investigating the effects of duty cycle on the outcome of CIA are essential to develop strategies to overcome the interpatient variability seen in current clinical VNS clinical trials of RA (Koopman et al., 2016; Genovese et al., 2020).

A limitation to this study was the relatively short experimental testing period, which resulted in significant tissue edema, synovitis and inflammatory infiltration but only mild bone damage, which is in line with other studies (Schopf et al., 2006). It is more common for arthritis treatment studies using the CIA model to allow the disease to progress for 21 days (Schopf et al., 2006). However, in earlier pilot trials of our CIA model, the disease severity in a small proportion of animals was severe and it was deemed unethical to go past 17 days. Furthermore, VNS was applied prior to the macroscopic onset of CIA and prevented the onset of moderate symptoms. This model is not representative of patients with drug-resistant RA and so future studies might consider alternative models, such as adjuvant-induced arthritis which manifest more severe bone erosion damage (Weichman, 1989).

In conclusion, abdominal VNS was efficacious in reducing arthritis severity in a rat model of CIA and supports the rationale for using abdominal VNS in patients with RA as a feasible alternative to the current approach of using cervical VNS. Intra-abdominal implantation surgery is admittedly more invasive than cervical vagus nerve implantation surgery and associated with a degree of risk (Ikramuddin et al., 2014). However, future clinical studies may demonstrate that a reduction in off-target effects and an increase in the therapeutic stimulation window may improve response rates to VNS therapy in patients with arthritis.

## Data availability statement

The raw data supporting the conclusions of this article will be made available by the authors, without undue reservation.

## Ethics statement

The animal study was reviewed and approved by the Bionics Institute Animal Research Ethics Committee (31-019) and complied with the Australian Code for the Care and Use of Animals for Scientific Purposes (National Health and Medical Research Council of Australia) and the Prevention of Cruelty to Animals (1986) Act.

## Author contributions

SCP was involved in conception and experimental design, acquisition and analysis of data, and was the primary writer of the manuscript. ER was a rheumatologist that provided intellectual feedback on experimental design and scored the histology (Figure 3). TH conducted all surgeries, prepared figures, and drafted significant parts of the manuscript. FM was a veterinarian specialized in histopathology and scored the histology (Figure 3). JBF was involved in conception and contributed to interpretation of results. All authors made substantial, direct, and intellectual contributions to the study and manuscript, and were involved in reviewing the manuscript.
